# Association of ABO blood groups with venous thrombosis recurrence in middle-aged patients: insights from a weighted Cox analysis dedicated to ambispective design

**DOI:** 10.1186/s12874-023-01915-7

**Published:** 2023-04-22

**Authors:** Gaëlle Munsch, Louisa Goumidi, Astrid van Hylckama Vlieg, Manal Ibrahim-Kosta, Maria Bruzelius, Jean-François Deleuze, Frits R. Rosendaal, Hélène Jacqmin-Gadda, Pierre-Emmanuel Morange, David-Alexandre Trégouët

**Affiliations:** 1 Univ. Bordeaux, Inserm, Bordeaux Population Health Research Center, UMR 1219, 33000 Bordeaux, France; 2 Cardiovascular and Nutrition Research Center (C2VN), INSERM, INRAE, Aix-Marseille University, Marseille, France; 3 Department of Clinical Epidemiology, Leiden University Medical Center, Leiden, Netherlands; 4 Department of Medicine Solna, Karolinska Institute, Stockholm, Sweden; 5 Department of Hematology, Karolinska University Hospital, Stockholm, Sweden; 6 Université Paris-Saclay, CEA, Centre National de Recherche en Génomique Humaine, 91057 Evry, France; 7 Centre d’Etude du Polymorphisme Humain, Fondation Jean Dausset, Paris, France

**Keywords:** Ambispective design, Survival analysis, Venous thrombosis, Recurrence, *ABO* blood groups, Genetic association studies

## Abstract

**Background:**

In studies of time-to-events, it is common to collect information about events that occurred before the inclusion in a prospective cohort. When the studied risk factors are independent of time, including both pre- and post-inclusion events in the analyses, generally referred to as relying on an ambispective design, increases the statistical power but may lead to a selection bias. In the field of venous thromboembolism (VT), *ABO* blood groups have been the subject of extensive research due to their substantial effect on VT risk. However, few studies have investigated their effect on the risk of VT recurrence. Motivated by the study of the association of genetically determined *ABO* blood groups with VT recurrence, we propose a methodology to include pre-inclusion events in the analysis of ambispective studies while avoiding the selection bias due to mortality.

**Methods:**

This work relies on two independent cohorts of VT patients, the French MARTHA study built on an ambispective design and the Dutch MEGA study built on a standard prospective design. For the analysis of the MARTHA study, a weighted Cox model was developed where weights were defined by the inverse of the survival probability at the time of data collection about the events. Thanks to the collection of information on the vital status of patients, we could estimate the survival probabilities using a delayed-entry Cox model on the death risk. Finally, results obtained in both studies were then meta-analysed.

**Results:**

In the combined sample totalling 2,752 patients including 993 recurrences, the A1 blood group has an increased risk (Hazard Ratio (HR) of 1.18, *p* = 4.2 × 10^–3^) compared with the O1 group, homogeneously in MARTHA and in MEGA. The same trend (HR = 1.19, *p* = 0.06) was observed for the less frequent A2 group.

**Conclusion:**

The proposed methodology increases the power of studies relying on an ambispective design which is frequent in epidemiologic studies about recurrent events. This approach allowed to clarify the association of *ABO* blood groups with the risk of VT recurrence. Besides, this methodology has an immediate field of application in the context of genome wide association studies.

**Supplementary Information:**

The online version contains supplementary material available at 10.1186/s12874-023-01915-7.

## Introduction

Venous Thromboembolism (VT) is a common cardiovascular disease with an annual incidence of ~ 1 to 3 per 1,000 in the general population which increases with age [1]. This pathology can manifest as either deep vein thrombosis (DVT) or pulmonary embolism (PE) with a mortality rate within a month of diagnosis at 6% and 12%, respectively [2].

After a first VT, the recurrence rate is approximately 30% within 10 years [3]. VT recurrence could be prevented by a continued anticoagulant treatment but this therapy leads to a substantial risk of bleeding and a significant cost to society [4]. Understanding the pathophysiological mechanisms of VT recurrence may facilitate the identification of groups of patients at lower risk of recurrence who do not require extended treatment. While more than 100 loci are now well established to be associated with the genetic susceptibility to VT [5–7], less is known about the genetic susceptibility to VT recurrence which possibly differs from that of first VT [8]. Among VT disease loci, the *ABO* locus, coding for the *ABO* blood groups, is one of the most important genetic risk factor due to the magnitude of the genetic effects associated with the A1 and B at-risk blood groups (Odds Ratio ~ 1.5) and their prevalence in the general population (18% and 8%, respectively) [9–12]. The attributable risk associated with *ABO* blood groups can be as high as 30% in some specific groups of individuals [13]. To date, few studies have explored the effect of *ABO* blood types on VT recurrence risk [14–17]. These analyses have generally been conducted in studies of moderate size with few recurrent events and have often relied on serological measurement of blood groups. Recently, our group showed that molecularly defined blood groups are more reliable than serological measurements [12]. In this work, we wish to investigate the effect of molecularly defined *ABO* blood groups on the risk of recurrence in two large VT cohorts, the MARTHA and MEGA studies [18, 19].

In MEGA, participants were included at the time of their first VT which represents the beginning of the at risk period for the recurrence. To study the risk of first recurrence a standard time-to-event analysis among which the Cox model is the most popular one [20] can be used. The MARTHA study has a different design since it included all subjects who visited a Thrombophilia centre in Marseille (France) between 1994 and 2012 and had a history of VT (possibly many years before inclusion). Information on recurrence post-inclusion was collected at a follow-up visit several years later but many participants had already experienced a VT recurrence at the time of inclusion.

In the literature, several choices have been proposed to deal with recurrent events occurring before the time of inclusion: i) analysing only first recurrent events that occurred post-inclusion, while discarding that experienced the event of interest (VT recurrence in our case) before their inclusion [21, 22], ii) analysing only recurrent events that occurred post-inclusion while stratifying according to the number of events before inclusion [23], iii) analysing all recurrent events that occurred post-inclusion without distinguishing between patients who did or did not experience a recurrence prior to inclusion [24].

Most of these approaches have been proposed to assess risk factors that are time-dependent variables such as biological measurements explaining why they only include the events occurring after the collection of the studied variables as the exposure must be measured before the event occurrence in order to avoid bias due to reverse causality. However, when the explanatory variables do not change over time, as genetic factors, this bias is avoided. When the dates of the events that occurred before the inclusion in the study are known, considering this information in the analysis could greatly increase the statistical power. However, specific data analysis procedures should be considered to avoid selection bias by death.

Therefore we here propose an original weighted survival analysis that enables the joint analysis of patients with or without recurrence prior inclusion in the study. This approach can be applied in any studies about recurrent events when information is collected about events that occurred before the inclusion and when the explanatory variables are time-independent, such as genetic factors. The proposed methodology is then employed to efficiently analyse the impact of *ABO* blood groups on first VT recurrence in MARTHA.

## Materials and methods

### MARTHA study

This work was motivated by the identification of genetic risk factors for VT recurrence in the MARseille THrombosis Association (MARTHA) study [25, 26]. MARTHA includes 2,837 unrelated VT patients who had a consultation visit at the Thrombophilia centre of La Timone Hospital in Marseille (France) between 1994 and 2012. The inclusion date of patients refers to this visit. All patients with at least one documented VT and free of any chronic conditions and of any well characterized genetic risk factors including homozygosity for Factor V Leiden or Factor II 20210A, protein C, protein S and antithrombin deficiencies, and lupus anticoagulant, were eligible. As an ancillary genetic study, a subsample of 1,592 MARTHA patients have been typed by a high density genotyping arrays, referred thereafter as the MARTHA GWAS subsample (where GWAS stands for Genome Wide Association Study) [27]. The MARTHA GWAS sub-study was further extended over the 2013–2018 period and patients were re-contacted to gather information on post-inclusion VT events.

### MARTHA GWAS sub-study and VT recurrence

The previous application of standard quality control procedures on the genome wide genotype data of MARTHA participants has led to the selection of 1,542 VT patients for genetic analyses [27]. From these remaining individuals, we further excluded patients with autoimmune disease or cancer at inclusion, or with missing information on time to VT recurrence for concerned patients. Finally, 1,518 VT patients were left for the VT recurrence analysis. Among these patients, 411 already had at least one VT recurrence before inclusion. The dates, types (DVT or PE) and provoked characters of the first VT and first recurrence were collected. During the 2013–2018 period, patients were re-contacted via phone call, mail questionnaire or medical visit, to gather information on post-inclusion VT. Among the 1,107 patients with a unique VT at inclusion, 846 (76%) could be re-contacted which led to the identification of 160 additional first recurrences. At the end of the second phase, information on the vital status of non-responders was obtained either through the Répertoire National d’Identification des Personnes Physiques (RNIPP) or medical data. Vital status was finally available for 1,380 individuals including 73 deaths.

### Ambispective design

**Fig. 1:**
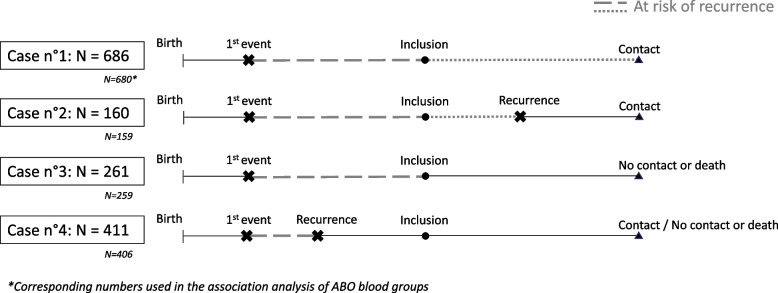
Illustration of the 4 scenarii of patients included in the MARTHA study. Case 1: patients who had a single VT before inclusion in the study and who were followed up during the recontact phase within which no recurrent event was observed. Case 2: patients who had a single VT before inclusion and experienced a recurrent event which was collected during the recontact phase. Case 3: patients with a single VT at inclusion for whom no follow-up information was collected during the recontact phase (i.e. lost to follow-up). Case 4: patients who had both a first VT and a recurrence before the inclusion in the study. For all 4 situations, the at-risk period is shown in grey with dotted lines for the post-inclusion period and with dashed lines for the retrospective period

As the current project aims to assess the effect of *ABO* polymorphisms on the risk of recurrence after a first VT, 4 different types of MARTHA participants can be distinguished (Fig. 1). Case 1 corresponds to patients who had a single VT before inclusion in the study and who were followed up during the recontact phase within which no recurrent event was observed. Case 2 represents patients who had a single VT before inclusion and experienced a recurrent event which was collected during the recontact phase. Case 3 corresponds to patients with a single VT before inclusion for whom no follow-up information was collected during the recontact phase (i.e. lost to follow-up). Finally, Case 4 represents patients who had both a first VT and a recurrence before the inclusion in the study. For each of these 4 situations, the at-risk period, a key element in the analysis of recurrent data that represents the period of time that contributes to the estimation of recurrence risk, is shown in grey in Fig. 1.

In a standard cohort analysis, only the post-inclusion period of patients from Cases 1&2 (represented with dotted lines), thereafter referred to as the “*prospective sample*”, would be used to investigate risk factors for recurrence. However, since genetic polymorphisms are fixed at birth, all cases of patients can contribute to the analysis of the genetic susceptibility of VT recurrence, considering the first VT as the starting point of the analysis (non-solid lines). This last comment also holds for non-genetic variables available at the time of first VT that are fixed over time such as sex and age at first VT. In the following, we will refer to the “*ambispective sample*” [28] when the four situations are simultaneously considered as it includes both pre- and post-inclusion VT recurrences, that is recurrences which occurred before or within the observation window.

Finally, the MARTHA *prospective sample* was composed of 846 patients including 160 VT recurrences and the extended *ambispective sample* involved 1,518 patients including 571 recurrent VT.

### Statistical modelling of recurrent events using weighted Cox model

The Cox proportional-hazards model is a popular semi-parametric model proposed by Cox in 1972 [29]. The relationship between the instantaneous risk function (or hazard function) associated with the occurrence of an event and the vector $Z$ of explanatory variables can be written as follows: ${\lambda \left(t,Z,\beta \right)= \lambda }_{0}\left(t\right)\mathrm{exp}({\beta }^{\top }Z)$ where $\beta$ is the vector of regression coefficients and ${\lambda }_{0}(t)$ represents the baseline hazard function.

In order to account for a possible selection bias due to mortality induced by the selection of MARTHA participants, we are proposing a weighted Cox analysis with weights defined by the inverse of the survival probability of individuals up to the time when the information on their possible recurrent event was collected. These weights are used to assign a higher weight to individuals who were less likely to be observed, e.g. individuals at high risk of death before collection of information on VT recurrence [30]. To estimate these weights, we had to model the risk of death in the MARTHA population of VT survivors, using the information on the vital status available for 1,380 patients. As age is the main risk factor for death, we estimated a delayed-entry Cox model with age as time-scale allowing a non-parametric modelling of the age effect. The delayed-entry Cox model allows to consider that individuals can be included in the study only if they are alive at the time of inclusion and thus they are at risk of death only from their age of entry into the study [31]. For this analysis, subjects contributed from their age of inclusion in the study to their death or last information on the vital status. Estimated parameters from this model were used to compute for all MARTHA patients their individual survival probabilities up to the appropriate time point according to their own clinical and covariates information. While for Cases 1&2 patients, the collection of information on VT recurrence is conditional to the survival of patients up to the recontact date, the collection of this information for Case 3&4 patients is conditional to their survival up to their inclusion in the study.

Once these weights are computed, they can be used in a weighted Cox model for the risk of first recurrence, with delay since the first VT as time scale, to analyse both *prospective* and *ambispective* samples. As the prospective analysis considers only post-inclusion events, a model with delayed-entry at the time of inclusion was estimated. This is not necessary for the *ambispective* analysis as all available information since the first VT is then considered. Once Hazard Ratio (HR) association parameters are obtained, their variance can be estimated using the robust method accounting for the within-subject correlation induced by the weights [32]. The weights were computed and standardized using the survival probabilities so that their sum corresponds to the studied sample size with the following formula:

$${w}_{i}=\frac{1/{s}_{i}}{\sum_{i=1}^{N}\frac{1}{{s}_{i}}}$$

With ${w}_{i}$ the weight of $i$^th^ individual, ${s}_{i}$ the survival probability of the $i$^th^ individual at its own data collection time and $N$ the studied sample size. This approach is implemented with the *survival* package of the R version 3.6.1 environment [33, 34].

### The MEGA study

Briefly, MEGA is a case–control study for VT that includes almost 4,900 patients who were included for their first VT between 1999 and 2004 [35]. Among them, 1,289 VT cases who were free of cancer and who provided a high-quality blood sample were eligible to have their DNA analysed and therefore had available genetic data as it has been previously described [20]. Between 2008 and 2009, questionnaires were sent to patients to gather information on a possible VT recurrence. From the 1,289 VT patients, we excluded 9 individuals who died before the re-contact phase, 17 individuals with missing information on the provoked character of the first VT event and 9 individuals who were homozygous for the factor V Leiden in order to match to MARTHA exclusion criteria. Six patients from the MEGA study (0.5%) were further excluded as it was not possible to unambiguously determine their *ABO* blood group (see next paragraph). Finally, 1,248 MEGA patients including 428 recurrences were included in the analysis. As these patients were included for their first event, a Cox model in which the delay since the first event was employed as time scale to investigate risk factors of first VT recurrence.

### *ABO* blood groups genetic determination

Five *ABO* polymorphisms were investigated in order to infer *ABO* blood groups. Following recent recommendations [12], the rs8176719-delG was used to tag for O1, the rs41302905-T allele for O2, the rs2519093-T for A1, the rs1053878-A for A2 and the rs8176743-T allele for B.

As MARTHA and MEGA participants have been typed by high-throughput genotyping arrays and imputed on the 1000G Phase I Integrated Release Version 2 Haplotypes, we used best-guessed genotypes from imputed data to infer *ABO* blood groups [27, 36]. Note that all 5 polymorphims have imputation quality greater than 0.9 in MARTHA and in MEGA. It was possible to infer *ABO* haplotypes and pair of haplotypes without ambiguity for 1,504 (99.1%) and 1,248 (99.5%) MARTHA and MEGA participants, respectively. Finally, the MARTHA *prospective sample* was composed of 839 individuals including 159 recurrences, the extended MARTHA *ambispective sample* included 1,504 among which 565 recurrences were observed and the MEGA sample was composed of 1,248 individuals including 428 recurrences. A detailed flow chart of the MARTHA sub- samples is presented in Supplementary Figure S1.

### Modelling strategy

Association of *ABO* blood groups with first recurrence was tested assuming additive effects of *ABO* tagging polymorphims, using the O1 group as a reference. Analyses were adjusted for sex, provoked status of the first VT (corresponding to the presence of a risk factor which temporarily promotes VT such as pregnancy or surgery), age at first VT, type of the first VT (DVT or PE) and the first 4 principal components derived from the GWAS genotypic data, in accordance with the literature [8].

Finally, *ABO* association parameters from the MARTHA *ambispective* and MEGA analyses were meta-analysed using a fixed-effects model (Mantel–Haenszel methodology) to highlight the observed trends [37].

## Results

### Population characteristics

The main characteristics of the MARTHA *prospective* and extended *ambispective* samples are shown in Table 1. MARTHA patients were included in the study at approximately 47 years old with a mean age at the first VT around 41. Patients were included in average 6 years after their first VT. The distribution of the age at enrolment and the delay between enrolment and the first VT are provided in Supplementary Figures S2 & S3. For approximately 80% of patients, the first VT was DVT and in two-thirds of individuals the first VT was provoked. One-third of patients were male, with a higher proportion of men in those with recurrences. On average, patients were followed for 9 years and this time was longer for patients without recurrence in both samples, since follow-up ends at the occurrence of a recurrence. Regarding *ABO* blood groups, O1 was the most frequent (~ 50%) followed by A1 (~ 33%), B (~ 9%), A2 (~ 6%) and finally O2 (~ 2%).

**Table 1:** Description of the main characteristics in the prospective and ambispective MARTHA samples

Variables	MARTHA *Prospective sample*			MARTHA *Ambispective sample*		
	**Total** ***N* = 839**	**Recurrences** ***N* = 159**	**Non-recurrences** ***N* = 680**	**Total** ***N* = 1,504**	**Recurrences** ***N* = 565**	**Non-recurrences** ***N* = 939**
	**N (%)**	**N (%)**	**N (%)**	**N (%)**	**N (%)**	**N (%)**
**Gender**						
Men	275 (32.8%)	63 (39.6%)	212 (31.2%)	509 (33.8%)	232 (41.1%)	277 (29.5%)
**Age at inclusion** (mean ± SD)	45.2 ± 14.9	44.0 ± 13.9	45.5 ± 15.4	47.1 ± 15.4	50.0 ± 14.7	45.3 ± 15.5
**Age at the first VT** (mean ± SD)	42.3 ± 15.5	41.5 ± 14.4	42.5 ± 15.8	41.0 ± 15.7	40.5 ± 15.1	41.3 ± 16.0
**Delay between inclusion and first VT** (In years, mean ± SD)	2.9 ± 6.2	2.5 ± 5.3	3.0 ± 6.4	6.1 ± 9.85	9.5 ± 11.3	4.0 ± 8.2
**Type of the first VT**						
DVT	653 (77.8%)	122 (76.7%)	531 (78.1%)	1,189 (79.1%)	454 (80.4%)	735 (78.3%)
**Characteristic of the first VT**						
Provoked	544 (64.8%)	93 (58.5%)	451 (66.3%)	993 (66.0%)	368 (65.1%)	625 (66.6%)
**Age at the collection of information on recurrence** (mean ± SD)^a^	54.9 ± 15.2	54.9 ± 13.9	54.9 ± 15.5	52.5 ± 15.6	43.0 ± 14.2	52.1 ± 16.4
**Delay of follow-up in years**^b^ (In years, mean ± SD)	8.8 ± 5.5	6.4 ± 5.3	9.4 ± 5.4	9.7 ± 9.6	7.9 ± 8.9	10.8 ± 9.8
**ABO haplotypes**						
A1	32.8%	37.4%	31.7%	33.5%	35.8%	32.2%
A2	5.5%	5.3%	5.5%	5.9%	6.9%	5.3%
O1	51.0%	47.5%	51.8%	49.8%	46.9%	51.5%
O2	1.7%	1.6%	1.7%	1.5%	1.5%	1.5%
B	9.1%	8.2%	9.3%	9.3%	8.9%	9.5%

^a^Since inclusion in the prospective sample, since the first VT in the ambispective sample

^b^Refers to the recontact for Cases 1 & 2 and inclusion for Cases 3 & 4 (see Fig. 1)

A description of the principal characteristics of the MEGA participants is provided in Table 2. The main differences with MARTHA sample are a higher proportion of men (49%), a higher age at first VT (~ 48yrs), a lower rate of DVT (61%) and a shorter (~ 5yrs) follow-up. The delay between inclusion and the first VT is not presented as only incident VT cases were recruited.

**Table 2:** Description of the main characteristics in the MEGA sample

Variables	MEGA *sample*		
	**Total** ***N* = 1,248**	**Recurrences** ***N* = 428**	**Non-recurrences** ***N* = 820**
	**N (%)**	**N (%)**	**N (%)**
**Gender**			
Men	661 (49.0%)	272 (63.6%)	389 (47.4%)
**Age at the first VT** (mean ± SD)	48.0 ± 12.8	49.8 ± 12.8	47.1 ± 12.8
**Type of the first VT**			
DVT	763 (61.1%)	270 (63.1%)	493 (60.1%)
**Characteristic of the first VT**			
Provoked	847 (67.9%)	237 (55.4%)	610 (74.4%)
**Delay of follow-up since inclusion** (In years, mean ± SD)	5.2 ± 2.9	3.1 ± 2.2	6.3 ± 2.7
**ABO haplotypes**			
A1	28.9%	31.8%	27.4%
A2	7.0%	7.7%	6.6%
O1	53.4%	50.9%	54.7%
O2	1.8%	1.5%	2.0%
B	8.9%	8.1%	9.3%

The sample used to estimate the risk of death in MARTHA was composed of 1,380 patients among whom 73 deaths were observed (Supplementary Table S1). The mean time of follow-up according to the last known vital status was around 12 years and other characteristics were similar to the MARTHA *ambispective* sample.

### Risk of death estimation

We estimated the risk of death in MARTHA with a delayed-entry Cox model (Supplementary Figure S4). The explanatory variables of this model were sex, provoked character of the first VT, age at the first VT and the first four principal components of the population stratification. Men had an HR (95% Confidence Interval) for death of 1.44 (0.87–2.40) whereas the provoked character of the first VT (HR = 0.42 (0.24–0.74)) and a higher age of first VT (HR = 0.98 (0.96–1.00) per year) were associated with reduced risk of death.

Using this model, we estimated the survival probability of patients up to the time at which information on their possible VT recurrence was collected. As described in *Methods* section, weights were based on survival probabilities and their range varied between 0.9 and 2.3 (Supplementary Figure S5).

### Clinical variables and VT recurrence risk

As a first step, we assessed the association of non-time dependent clinical variables on the risk of first VT recurrence in the MARTHA *prospective* and *ambispective* samples (Table 3). In the *prospective* analysis of 839 subjects including 159 recurrences, male sex was associated with an increased risk of VT recurrence (HR = 1.47 (1.03–2.09)). Other variables were not significantly associated with recurrence, but a trend was observed for the provoked character of the first VT that tends to be protective (HR = 0.69 (0.48–1.00)).

**Table 3:** Association of clinical variables and *ABO* haplotypes with VT recurrence in MARTHA (prospective and ambispective) and MEGA

Variables	MARTHA *Prospective*		MARTHA *Ambispective*		MEGA		Meta-analysis MARTHA Ambispective & MEGA	
	***N* = 839** **Nb recurrences = 159**		***N* = 1,504** **Nb recurrences = 565**		***N* = 1,248** **Nb recurrences = 428**			
	**HR (95% CI)**	***P***	**HR (95% CI)**	***P***	**HR (95% CI)**	***P***	**HR (95% CI)**	***P***
**Gender**								
Men	1.47 (1.03–2.09)	0.034	1.65 (1.36–2.01)	4.0 × 10^–7^	1.81 (1.46–2.25)	5.9 × 10^–8^	1.72 (1.47–2.01)	3.0 × 10^–12^
**Age at the first VT** (10 years increase)	0.91 (0.81–1.02)	0.105	1.08 (1.02–1.15)	0.020	0.99 (0.92–1.07)	0.810	1.05 (0.99–1.11)	0.107
**Type of the first VT**								
DVT	0.85 (0.60–1.21)	0.368	1.17 (0.96–1.42)	0.140	1.15 (0.95–1.40)	0.160	1.16 (1.01–1.33)	0.036
**Characteristic of the first VT**								
Provoked	0.69 (0.48–1.00)	0.059	0.99 (0.80–1.23)	0.920	0.61 (0.49–0.76)	6.7 × 10^–6^	0.78 (0.67–0.91)	1.2 × 10^–3^
**ABO haplotypes**								
A1	1.32 (1.02–1.70)	0.035	1.15 (1.00–1.32)	0.045	1.21 (1.03–1.42)	0.018	1.18 (1.05–1.33)	4.2 × 10^–3^
A2	1.13 (0.68–1.88)	0.644	1.27 (0.98–1.64)	0.061	1.11 (0.86–1.43)	0.409	1.19 (1.00–1.42)	0.062
O1	Reference		Reference		Reference		Reference	
O2	1.05 (0.47–2.35)	0.896	1.19 (0.73–1.94)	0.476	0.86 (0.50–1.49)	0.584	1.03 (0.70–1.52)	0.880
B	1.00 (0.64–1.57)	0.998	1.02 (0.82–1.27)	0.874	1.00 (0.78–1.29)	0.987	1.01 (0.85–1.21)	0.900

*HR* Hazard Ratio, *CI* Confidence Interval

The analysis of the same variables performed in the extended *ambispective* sample allows to refine some of these observations with a higher power. Male sex was still associated with a higher risk of recurrence HR = 1.65 (1.36–2.01); and older age at first VT appeared as deleterious (HR = 1.08 (1.02–1.15) for a 10 years increase). Conversely, we did not find any trend for the provoked status of the first VT (HR = 0.99 (0.80–1.23)).

In MEGA, male sex was also associated with an increased risk of recurrence (HR = 1.81) but older age was not. Besides, the provoked status of the first VT was significantly associated with a decreased risk of recurrence (HR = 0.61), as initially observed in the MARTHA *prospective* analysis but not confirmed in the *ambispective* analysis.

Lastly, even if the type of first VT (DVT vs PE) was not significantly associated with VT recurrence in neither of the two studies, the same trend for a higher risk of recurrence associated with DVT was observed in the MARTHA *ambispective* (HR = 1.17) and the MEGA (HR = 1.15) samples. The meta-analysis of these two HRs yielded a combined HR of 1.16.

### *ABO* blood groups

In the MARTHA *prospective* sample, we observed a significant association of A1 blood group compared with O1 on the risk of first VT recurrence (HR = 1.32 (1.02–1.70); *p* = 0.035) which was confirmed in the analysis of the *ambispective* sample (HR = 1.15 (1.00–1.32); *p* = 0.045) (Table 3). The same trend was observed for the A2 group but did not reach statistical significance (HR = 1.27 (0.98–1.64); *p* = 0.061 in the *ambispective* analysis). In MEGA, only A1 was significantly associated with a higher risk of VT recurrence (HR = 1.21; *p* = 0.018). No evidence for association with VT recurrence was observed for B and O2 groups in either MARTHA or MEGA.

Finally, based on the meta-analysis of the results observed in the MARTHA *ambispective* and MEGA samples, the risk of VT recurrence associated with *ABO* blood groups compared with O1 were HR = 1.18 (*p* = 4.2 × 10^–3^), HR = 1.19 (*p* = 0.06), HR = 1.01 (*p* = 0.90) and HR = 1.03 (*p* = 0.88) for A1, A2, B and O2, respectively.

Since MARTHA *ambispective* and MEGA samples slightly differed with respect to the proportion of DVT events at first VT, the age at first VT, and the delay of follow-up (Tables 2 & 3), we further assessed whether the observed association of *ABO* blood groups was consistent according to these variables. No evidence for heterogeneity was observed whether for type of first VT (Supplementary Table S2), for age at first VT (Supplementary Table S3) or delay of follow-up (Supplementary Table S4).

## Discussion

The motivation of this work was to investigate the risk of VT recurrence associated with *ABO* blood groups in two large cohorts of middle-aged VT patients, MARTHA and MEGA, the former being built upon an ambispective design.

In order to maximize the power of the MARTHA study where about 70% of first VT recurrences occurred in patients before their inclusion in the study, we developed a weighted approach to analyse non-time dependent risk factors (such as genetic polymorphisms) of an event which could have occurred in patients before their inclusion in the study. Such recurrent events are generally discarded in standard approaches that focus only on recurrent events occurring post-inclusion [21–24]. Our proposed modelling relies on a weighted Cox model where the use of weights allows to limit the selection bias associated with the use of pre-inclusion events and thus to gain statistical power by jointly analysing pre- and post-inclusion recurrent events. This method differs from the weighting approach for repeated events proposed to deal with event-dependent sampling [38]. Indeed, the inclusion in MARTHA depends on an event, the first VT, which is not the outcome of interest; the first VT defines the beginning of the period at risk for the recurrence. Our weighting approach handles potential bias due to mortality until the time of data collection for the recurrence.

Our proposed weighted estimation approach is unbiased if the weights are well-specified which means that the Cox model for death is correct. In this work, the death model has two main limitations. First, as the information on VT recurrence was often missing for subjects who died, it was not possible to include VT recurrence (and other possible unknown variables) as a risk factor in our model for death risk. Second, we assumed the proportionality of the risk of death and did not account for the calendar time that could modify either the baseline risk of death or the association with risk factors for death. Moreover, as the number of death during the follow-up in MARTHA is quite small, a Monte Carlo analysis was performed to evaluate the sensitivity of the results to the uncertainty on the weights (description is available in the Supplement). Despite some slight variability in HRs’ estimates (especially for O2 group), the overall results remain unchanged.

In this work, we were interested in the association of *ABO* blood groups with the risk of first VT recurrence and not with the risk of multiple VT recurrences. Indeed, at inclusion in MARTHA, only detailed information on first VT and possibly first recurrence was collected, preventing us from investigating the association with multiple recurrences. Besides, the analysis of such multiple events would require more complex modeling that would take into account the correlation of repeated events [39].

The analysis of these two studies, totaling 2,752 VT patients including 993 recurrences, revealed that the A1 and A2 blood groups were both associated with a moderate increased risk of VT recurrence, HR ~ 1.20 for both, compared with O1. Note that, likely because of the modest frequency of the A2 blood group (~ 5%), the association was only marginally significant (*p* = 0.06). Some studies have already investigated the association of *ABO* blood groups with the risk of VT recurrence [14–17], but often with a moderate sample size or using serological *ABO* phenotypes whereas we here used genetically defined *ABO* blood groups which has been shown to be more efficient to capture the effect of *ABO* on VT risk [12]. Our results are consistent with those showing a higher risk of recurrence in non-OO patients [15–17]. However, they are discordant with the study of *Baudouy* *et al.* who found a higher risk of VT recurrence in B blood group patients [14], while no association (HR = 1.01, *p* = 0.90) was observed in our work. This lack of association in our work is unlikely due to a power issue as the B blood group was more frequent than A2 which was significantly associated here with VT recurrence. Beyond its rather modest size (*N* = 100) and the analysis of serological *ABO* phenotypes, the work of *Baudouy* *et al.* focused on patients with PE as first VT. A stratified analysis of *ABO* blood groups with recurrence according to the type of the first event (DVT or PE) did not reveal in our work any evidence for specific sub-group *ABO* effects (Supplementary Table S2).

MARTHA and MEGA are composed of middle-aged VT patients, with average age at first VT event ~ 45 yrs. While the association of *ABO* blood groups with VT recurrence was consistent between patients with age at first event lower and higher than 45yrs (Supplementary Table S3), our study is not well-suited to assess whether the observed *ABO* association also holds in older ages. Our results cannot then be generalizable to older populations and further studies are mandatory to investigate this issue.

As the proposed *ambispective* modelling is only valid for analysing non-time dependent variables, we could not adjust the *ABO* blood group‘s effect on biological variables that have only been measured at the time of inclusion, such as von Willebrand Factor (vWF). Adjusting for vWF plasma levels could have allowed us to assess whether *ABO* blood groups impact on VT recurrence independently of vWF, at least partially. This is plausible as the observed pattern of association of *ABO* blood groups with recurrence does not match the known associations between *ABO* blood groups and vWF plasma levels [12]. The observed pattern of association with recurrence does not match either the known associations with first VT risk. Indeed, the B blood group did not show any trend for association with VT recurrence while it is associated with an Odds Ratio of ~ 1.5 for first VT [12]. Interestingly, the observed pattern matches with the one observed between *ABO* blood groups and plasma levels of Intercellular Adhesion Molecule 1 (ICAM1) where both A1 and A2 groups associate with ICAM1 levels, but not B [12]. These observations suggest that the biological factors involved in the association of *ABO* blood groups with VT recurrence differ from those involved in their relation with incident VT. More than 50 plasma proteins have been shown to be under the genetic influence of the *ABO* locus [40]. Determining which of them are associated with the risk of incident and/or recurrent VT merit further deep investigations. Finally, we could not adjust our analysis for the familial history of VT as the available information in MARTHA refers to the presence of a history at the time of inclusion and many recurrences arose earlier.

Nevertheless, our modelling enabled us to assess the impact on the risk of VT recurrence of several clinical variables that are fixed after the first VT event such as age at first VT and the type of first VT (DVT vs PE; provoked vs unprovoked). Consistent in MARTHA and in MEGA were the associations of male sex and DVT as first VT with an increased risk of first VT recurrence, confirming previous observations [8, 41]. However, we did not observe consistent results with respect to age at first VT nor with the provoked status of the first VT. For the effect of the provoked character on VT recurrence, the different trends observed can be due to the different design and sample selection between MARTHA and MEGA [42, 43]. Indeed, participants in MARTHA were included for at least one previous VT which may have occurred more than fifty years before their inclusion (Mean = 6 years; Standard Error = 10 years) whereas in MEGA, patients were recruited at the time of their first VT. Besides, the definition of the provoked character slightly differs between MARTHA and MEGA (Supplementary Table S5). We also observed some differences between MARTHA *prospective* and *ambispective* that might be due to the calendar time which has not been taken into account in our work. We are aware that the differences in the management, prevention and identification of VT events may have masked the association between the provoked character of the first event and VT recurrence in the *ambispective* analysis. Indeed, among the 25% of MARTHA patients that had their first VT before the start of the study (1994), for 80% of them the VT was provoked. Whereas in the remaining sample of 75% of MARTHA patients, the first VT was provoked in only 62% of cases. Of note, when we restrict the analysis to recurrent events that occur within less than 2 years after the first event, the provoked status of first VT was protective against recurrence, consistently in MARTHA and MEGA (Supplementary Table S4). These results are in line with previous findings from a 2-years follow-up study [42]. Furthermore, the association between A1 and A2 blood groups with VT recurrence remain unchanged when focusing on patients whose first VT occurred after the start of the MARTHA study. Altogether, we feel that such differences may have modest impact when one is interested in genetic factors as illustrated here with the consistent patterns observed for *ABO* blood groups in both MARTHA *prospective* and *ambispective* samples as well as in MEGA (Supplementary Table S6).

## Conclusion

This study demonstrated that both A1 and A2 blood groups are associated with increased risk of VT recurrence in middle-aged patients. This finding was made possible thanks to a new weighting approach that make possible to include events arising before the inclusion in addition to post-inclusion events in survival analyses considering only time independent risk factors. This approach that increases the study power, finds an immediate field of application to genetic association studies for time-to-events in cohorts where follow-up information for deaths is available and events before inclusion are collected. Directions for future research include more flexible modeling of the risk of death for estimating the weights (possibly considering non-proportionality, calendar time-trend and effect of multiple recurrences) and the extension of the weighted approach to models for multiple events.

## Supplementary Information

**Additional file 1.**

**Additional file 2.**

## Data Availability

Summary statistic of the data analyzed in this work are all provided in the main manuscript document and its supplements.

## Abbreviations

DVT: Deep Vein Thrombosis
GWAS: Genome Wide Association Study
HR: Hazard Ratio
ICAM1: Intercellular Adhesion Molecule 1
PE: Pulmonary Embolism
RNIPP: Répertoire National d’Identification des Personnes Physiques
VT: Venous Thromboembolism
vWF: Von Willebrand Factor
