# Supplementary material for: Association of ABO blood groups with venous thrombosis recurrence in middle-aged patients: insights from a weighted Cox analysis dedicated to ambispective design
Source: BMC Med Res Methodol. 2023 Apr 22;23:99. doi: 10.1186/s12874-023-01915-7 (PMC10122291; doi:10.1186/s12874-023-01915-7)
Supplement: Supplementary file 1 — Additional file 1. [file 12874_2023_1915_MOESM1_ESM.pdf]

Supplementary Materials

Association of ABO blood groups with venous thrombosis recurrence in middle-aged patients: insights from a weighted Cox analysis dedicated to ambispective design

Gaëlle Munsch, Louisa Goumidi, Astrid van Hylckama Vlieg, Manal Ibrahim-Kosta, Maria Bruzelius, Jean-François Deleuze, Frits R. Rosendaal, Hélène Jacqmin-Gadda, Pierre-Emmanuel Morange, David-Alexandre Trégouët

Contents:

Supplementary Figures 1-6 2

Supplementary Tables 1-3,5-6 5

Supplementary Text 10

Supplementary Figure S1. Flow chart of the MARTHA sub-samples

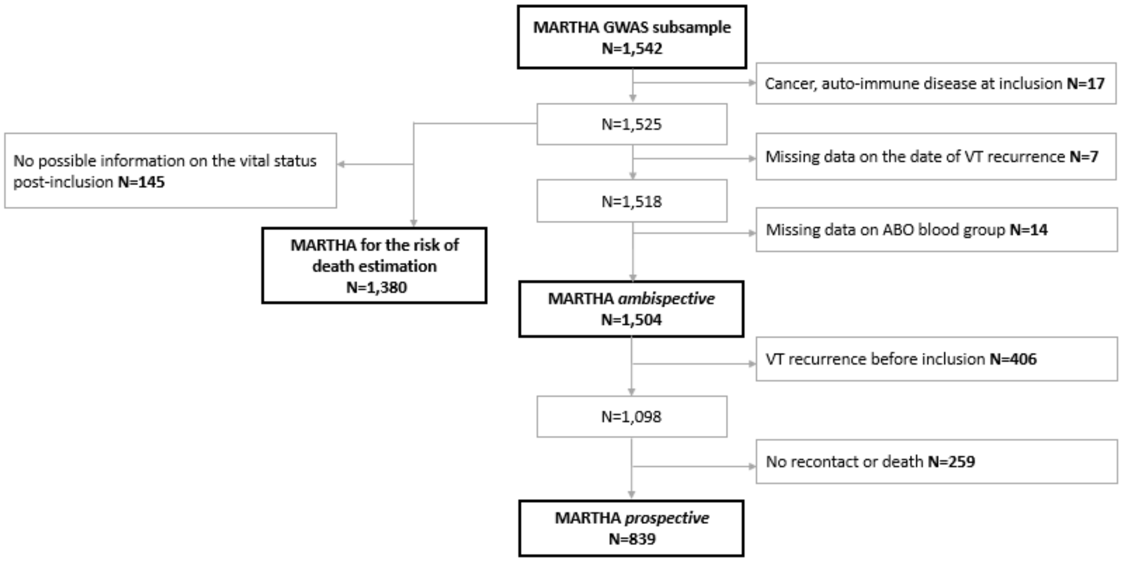

Supplementary Figure S2. Distribution of the age at enrolment in MARTHA participants (N=1,504)

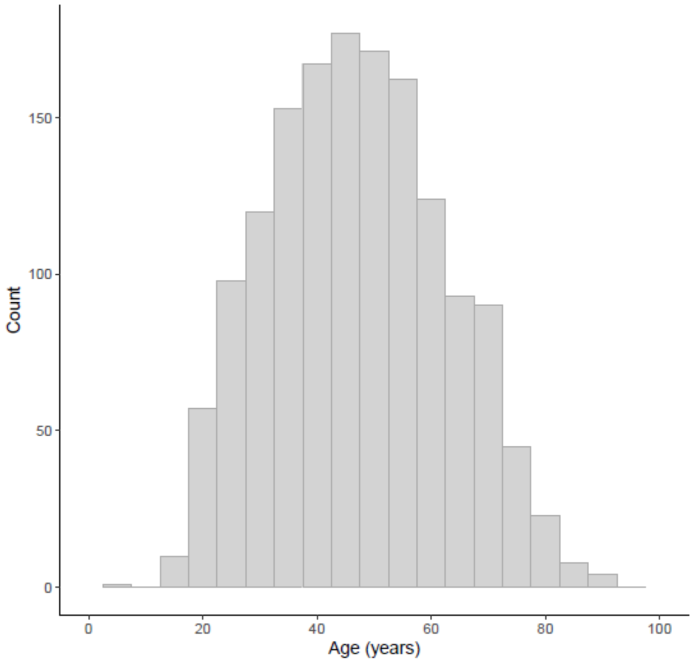

Supplementary Figure S3. Distribution of the delay between enrolment and the first VT in MARTHA participants (N=1,504)

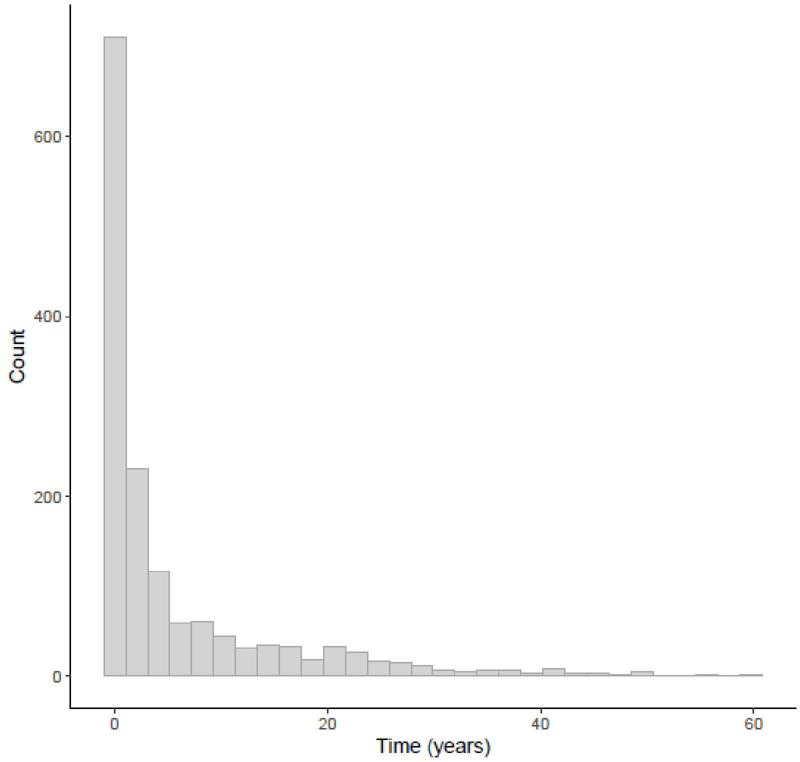

Supplementary Figure S4. Kaplan Meier plot of the survival probability in MARTHA participants with an available follow up (N=1,380 including 73 deaths)

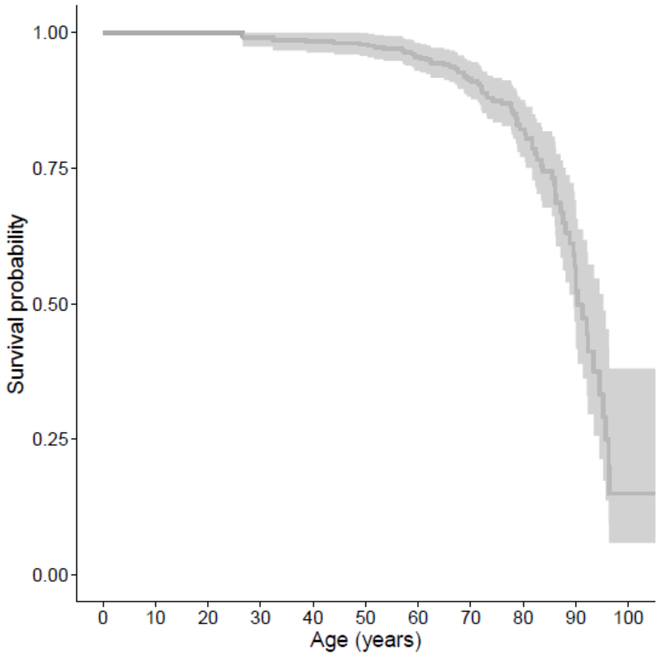

Supplementary Figure S5. Distribution of the estimated weights for the MARTHA participants (N=1,504)

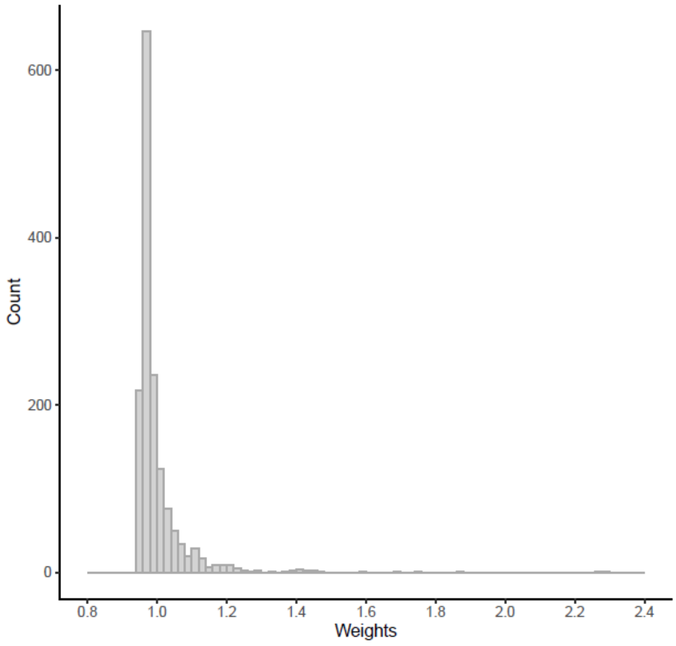

Supplementary Figure S6. Sensitivity of the association of *ABO* blood groups with recurrence according to the weights estimation in MARTHA (N=1,504)

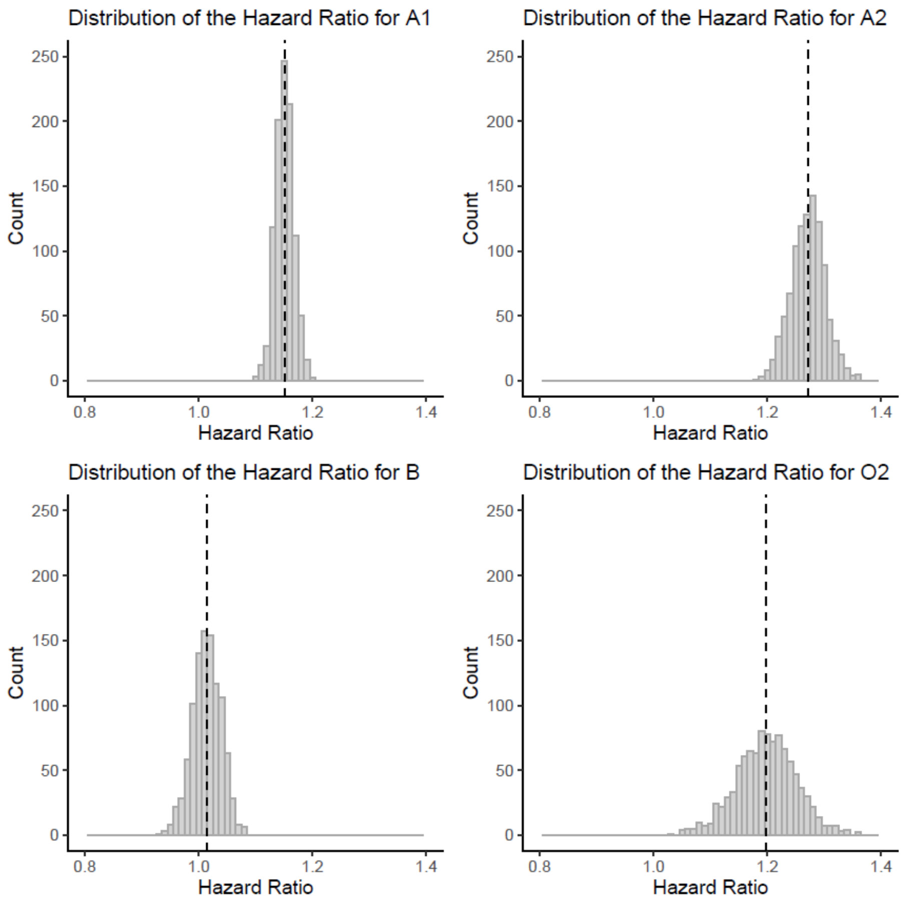

Note: The 4 panels show the distribution of the Hazard Ratio in the Monte Carlo resampling analysis (See Supplementary Text). The dashed line corresponds to the estimated value in the initial model

Supplementary Table S1. Description of the MARTHA sample for the death risk estimation

Variables	Total N=1,380 N (%)
**Gender**	
Men	468 (33.9%)
**Age at inclusion** (mean ± Standard Deviation (SD))	47.1 ± 15.3
**Age at the first VT** (mean ± SD)	41.3 ± 15.7
**Delay between inclusion and first VT** (In years, mean ± SD)	5.8 ± 9.6
**Type of the first VT**	
DVT only	1,087 (78.8%)
**Characteristic of the first VT**	
Provoked	911 (66.0%)
**Delay of follow-up in years*** (In years, mean ± SD)	11.8 ± 5.3

*According to the death event

Supplementary Table S2. Association of *ABO* haplotypes with first VT recurrence in MARTHA *ambispective* and MEGA stratified on the type of the first VT

Variables	MARTHA *Ambispective* N=1,504 Nb recurrences=565		MEGA N=1,248 Nb recurrences=428		Meta-Analysis Fixed-effects	
	**HR (95% CI)**	**P**	**HR (95% CI)**	**P**	**HR (95% CI)**	**P**
**ABO haplotypes – PE as first VT**	**N=315 ; 111 recurrences**		**N=485 ; 158 recurrences**			
A1	1.10 (0.82-1.48)	0.536	1.38 (1.05-1.82)	0.020	1.24 (1.02-1.51)	0.029
A2	1.87 (1.04-3.37)	0.039	0.79 (0.49-1.27)	0.329	1.11 (0.80-1.55)	0.554
O1	Reference		Reference		Reference	
O2	0.65 (0.13-3.24)	0.600	0.70 (0.28-1.72)	0.434	0.69 (0.36-1.32)	0.250
B	0.85 (0.47-1.53)	0.574	0.83 (0.51-1.36)	0.447	0.84 (0.59-1.20)	0.318
**ABO haplotypes – DVT as first VT**	**N=1,189 ; 454 recurrences**		**N=763 ; 270 recurrences**			
A1	1.16 (0.99-1.36)	0.063	1.14 (0.94-1.39)	0.211	1.15 (1.00-1.32)	0.045
A2	1.20 (0.91-1.58)	0.180	1.29 (0.94-1.77)	0.104	1.24 (1.00-1.54)	0.059
O1	Reference		Reference		Reference	
O2	1.41 (0.85-2.35)	0.180	0.94 (0.46-1.90)	0.872	1.23 (0.75-2.01)	0.422
B	1.06 (0.84-1.34)	0.612	1.08 (0.79-1.48)	0.637	1.07 (0.86-1.33)	0.566

HR: Hazard Ratio

CI: Confidence Interval

Supplementary Table S3. Association of *ABO* haplotypes with first VT recurrence in MARTHA *ambispective* and MEGA stratified on age at the first VT

**Variables**	**MARTHA *Ambispective*** **N=1,504** **Nb recurrences=565**		**MEGA** **N=1,248** **Nb recurrences=428**		**Meta-Analysis Fixed-effects**	
	**HR (95% CI)**	**P**	**HR (95% CI)**	**P**	**HR (95% CI)**	**P**
**ABO haplotypes – First VT before 45 years**	**N=932 ; 349 recurrences**		**N=487 ; 144 recurrences**			
A1	1.12 (0.94-1.33)	0.201	1.31 (0.99-1.72)	0.055	1.17 (0.97-1.42)	0.109
A2	1.33 (0.95-1.87)	0.098	1.44 (0.94-2.22)	0.098	1.37 (1.01-1.86)	0.043
O1	Reference		Reference		Reference	
O2	1.58 (0.85-2.93)	0.148	0.80 (0.29-2.20)	0.673	1.31 (0.64-2.77)	0.454
B	1.13 (0.88-1.44)	0.344	0.81 (0.50-1.33)	0.411	1.06 (0.75-1.49)	0.762
**ABO haplotypes – First VT after 45 years**	**N=572 ; 216 recurrences**		**N=761 ; 284 recurrences**			
A1	1.21 (0.97-1.50)	0.091	1.17 (0.96-1.42)	0.129	1.19 (1.03-1.36)	0.018
A2	1.21 (0.84-1.75)	0.310	1.00 (0.72-1.38)	0.999	1.09 (0.87-1.36)	0.476
O1	Reference		Reference		Reference	
O2	0.78 (0.36-1.71)	0.537	0.87 (0.44-1.71)	0.689	0.83 (0.52-1.34)	0.448
B	0.84 (0.55-1.29)	0.430	1.12 (0.82-1.53)	0.485	1.01 (0.81-1.26)	0.921

HR: Hazard Ratio

CI: Confidence Interval

Supplementary Table S5. Definition of the provoked character in MARTHA and MEGA

MARTHA study	MEGA study
• Surgery within 3 months before VT	• Surgery within 3 months before VT
• Pregnancy/ puerperium within 3 months before VT	• Pregnancy/ puerperium within 3 months before VT
• Oral contraceptive use within 3 months before VT	• Hormone use at the time of VT, including: hormone replacement therapy and hormonal contraceptives
	• Plaster cast within 3 months before VT
• Immobilization for 3 days or more within 3 months before VT	• Immobility in bed, in hospital: Confinement to bed ≥ 3 days in hospital, confinement to bed ≥ 3 days at home, within 3 months before VT
• Long travel (by car >10 hours ; by plane > 5 hours) within 3 months before VT	• Prolonged travel >4 hours within 2 months before VT
• Trauma of the lower limb within 3 months before VT	• Leg injury in 3 months before VT
• Pneumonia at time of VT	• Pneumonia in year before VT
• Infection at time of VT (urinary tract infection, pyelonephritis, arthritis, bursitis, sinusitis, pulpitis, inflammation elsewhere, hepatitis A, B or C)	• Infection in year before VT (urinary tract infection, pyelonephritis, arthritis, bursitis, sinusitis, pulpitis, inflammation elsewhere, hepatitis A, B or C)

Supplementary Table S6. Association of *ABO* haplotypes with first VT recurrence in MARTHA *ambispective* and MEGA stratified on the provoked character of the first VT

**Variables**	**MARTHA *Ambispective* N=1,504 Nb recurrences=565**		**MEGA N=1,248 Nb recurrences=428**		**Meta-Analysis Fixed-effects**	
	**HR (95% CI)**	**P**	**HR (95% CI)**	**P**	**HR (95% CI)**	**P**
**ABO haplotypes – First VT provoked**	**N=993 ; 368 recurrences**		**N=847 ; 237 recurrences**			
A1	1.16 (0.98-1.37)	0.072	1.22 (0.98-1.51)	0.070	1.18 (1.02-1.38)	0.029
A2	1.32 (0.94-1.86)	0.105	1.30 (0.93-1.82)	0.120	1.31 (1.04-1.66)	0.025
O1	Reference		Reference		Reference	
O2	0.97 (0.45-2.11)	0.940	0.66 (0.25-1.79)	0.420	0.84 (0.42-1.70)	0.627
B	1.15 (0.89-1.48)	0.289	0.93 (0.65-1.32)	0.670	1.07 (0.83-1.37)	0.615
**ABO haplotypes – First VT unprovoked**	**N=511 ; 197 recurrences**		**N=401 ; 191 recurrences**			
A1	1.17 (0.91-1.51)	0.213	1.23 (0.97-1.57)	0.094	1.20 (1.01-1.43)	0.037
A2	1.24 (0.86-1.78)	0.248	0.91 (0.60-1.37)	0.642	1.08 (0.81-1.44)	0.602
O1	Reference		Reference		Reference	
O2	1.41 (0.74-2.68)	0.291	1.09 (0.74-1.62)	0.667	1.22 (0.75-1.98)	0.431
B	0.82 (0.55-1.22)	0.325	1.02 (0.52-2.03)	0.947	0.95 (0.72-1.25)	0.698

HR: Hazard Ratio

CI: Confidence Interval

Supplementary Text. Sensitivity analysis on the weights estimation for MARTHA participants

Methods: To investigate the variability of the weights estimated from the MARTHA study and their impact on the weighted Cox model, we used a Monte Carlo method. From the death risk model, we estimated the survival function $\hat{S}(t_i|Z_i) = \exp(-\hat{A}(t_i|Z_i))$ of each individual i up to the time t_i (which corresponds to the time of collection of the information on VT recurrence). Assuming that the cumulative risk $\hat{A}(t_i|Z_i)$ follows a normal distribution, for each individual we randomly draw 1,000 values of the his/her cumulative risk from the distributions $N(\hat{A}(t_i|Z_i), SE(\hat{A}(t_i|Z_i)))$ and computed the corresponding survival probabilities $\hat{S}_k(t_i|Z_i) = \exp(-\hat{A}_k(t_i|Z_i))$ to obtain the set of individual weights w_{ik} for k=1,…,1000. Then 1,000 weighted Cox model for the VT recurrence were estimated.

Results: The distributions of the HR for the ABO blood groups from the 1,000 models for VT recurrence are shown in Supplementary Figure 6 where the value of the HR estimated in the initial model is presented as a dashed line. The empirical distributions are well centred at the initial estimated HRs and, for both A1 and A2, all estimated HRs are above 1 supporting our conclusions.
